# Influence of discrimination perception on career exploration of higher vocational students: Chain mediating effect test

**DOI:** 10.3389/fpsyg.2022.968032

**Published:** 2022-07-27

**Authors:** Xuejun Liu, Xianjun Sun, Qin Hao

**Affiliations:** ^1^School of Education Science, Nanjing Normal University, Nanjing, China; ^2^School of International Education, Shanghai University, Shanghai, China; ^3^School of Geography, Nanjing Normal University, Nanjing, China

**Keywords:** career exploration, core self-evaluation, psychological flexibility, discrimination perception, higher vocational students

## Abstract

Explore the influence mechanism of discrimination perception on higher vocational Students’ career exploration, it provides empirical evidence for promoting vocational college Students’ career exploration and career development. Using the questionnaire survey method, 893 higher vocational students from four higher vocational colleges in Jiangsu Province were investigated by using the Discrimination Perception Scale, the Core Self-Evaluation Scale (CSES), the Chinese version of the Acceptance and Action Questionnaire, the Chinese version of the Cognitive Fusion Questionnaire (CFQ) and the Career Exploration Scale (CES). The data were analyzed using SPSS26.0 and Amos23.0, and the results showed that discrimination perception was significantly negatively correlated with core self-evaluation, psychological flexibility and career exploration (*r* = −0.487, −0.497, −0.326, *p* < 0.01), core self-evaluation was significantly positively correlated with psychological flexibility and career exploration (*r* = 0.518, 0.352, *p* < 0.01), and psychological flexibility was significantly positively correlated with career exploration (*r* = 0.386, *p* < 0.01); Core self-evaluation and psychological flexibility mediated the effect between discrimination perception and career exploration with effect sizes of −0.054 and −0.061, respectively; Core self-evaluation and psychological flexibility mediated the chain effect between discrimination perception and career exploration of higher vocational students with effect sizes of −0.030. Therefore, discrimination perception not only directly influences career exploration of higher vocational students, but also indirectly influences career exploration of higher vocational students through the separate mediating effects of core self-evaluation and psychological flexibility and the chain mediating effects of core self-evaluation and psychological flexibility. Accordingly, the following suggestions are put forward, which should pay attention to the cultivation of core self-evaluation and psychological flexibility of higher vocational students, strengthen the teaching quality and improve the image of vocational colleges.

## Introduction

Career development theory divides personal career development into five stages: growth, exploration, establishment, maintenance, and decline (Super and Jordaan, 1973; Jordaan, 1977). Vocational college students are between the ages of 15 and 24, and are in the exploratory stage of career development (Chen et al., 2021). Career exploration is a mental or physical activity undertaken by an individual to achieve career goals that includes both information seeking and awareness of self and environment so that the individual has a clearer orientation to the establishment of future career development goals (Super and Knasel, 1981; Pavlova and Silbereisen, 2014). Several studies have shown that career exploration has a significant role in promoting individual career behaviors and facilitating career competence (Chan, 2018; Hu et al., 2020; Chen H. et al., 2022). Regarding the factors influencing career exploration, existing research has focused on family and individual factors (Selleck-Harwell, 2004). Family factors refer to family economic status, parents’ education level, and parenting style, while individual factors mainly include gender, personality, and career self-efficacy (Lindstrom et al., 2007; Wang et al., 2019). People have social attributes, and this study explores the influence mechanism of career exploration of higher vocational students from social issues.

Different from general education, vocational education is employment-oriented education. Vocational education helps educated people develop or improve the knowledge, skills, abilities, and other professional qualities required for a particular occupation. According to the theory of human capital, general education is “general human capital,” which is transferable and can play a role in different work. Vocational education is “specific Human Capital,” used to help workers adapt to specific jobs and make them more productive, both are important (Tilak, 2003). But in many developing countries, choosing vocational education does not seem to be a good choice for future development. Enterprises prefer to employ students with general education, believing that students with general education are more capable in work and study than those with vocational education. Such potential employment discrimination affects the self-confidence and enthusiasm of vocational college students in job hunting, and is not conducive to the active career exploration of vocational college students. According to social exclusion theory, labor market exclusion and social relationship exclusion interact with each other (Atkinson and Hills, 1998). The phenomenon of employment discrimination has a negative impact on the social interaction of students in higher vocational colleges. Discrimination perception is a subjective experience that individuals perceive that they are treated unfairly by others because of their membership in a group to which they belong (Yang et al., 2019). When individuals perceive external discrimination and have high levels of discrimination perception, their psychological wellbeing, work attitudes, organizational commitment, and career development are lower (Valentine et al., 1999; Triana et al., 2010; Valdivia and Flores, 2012; Harnois, 2015). In addition, in the era of increasing technological level, the substitution effect of technological progress on labor force is gradually emerging (Blien and Sanner, 2014). Many people lost their jobs during the COVID-19 lockdown (Harris and Samuel, 2020). The outbreak of the COVID19 pandemic has increased the unemployment rate in many parts of the world. From March to April 2020, the unemployment rate in the United States rose from 4.4 percent to more than 14.7 percent, and in Australia it rose from 5.4 percent to 11.7 percent (Suomi et al., 2020). Therefore, under the influence of multiple factors such as employment discrimination, the annual increase in the number of college graduates, the rapid development of science and technology and the COVID-19 pandemic, the employment situation of graduates from higher vocational colleges in many developing countries has become more and more severe.

Career exploration has a significant role in promoting individual career behaviors and facilitating career competence (Chan, 2018; Hu et al., 2020; Chen H. et al., 2022). Therefore, it is important to study the role of discrimination perception on higher vocational Students’ career exploration and the inner influence mechanism to enhance the employment competitiveness of this group and improve graduates’ employment problems.

## Research theory and hypothesis

### The mediating effect of core self-evaluation between discrimination perception and career exploration

Vocational education cultivates a large number of students with high professional quality and actively carry out vocational exploration activities. It has been proven that the injustice in the job market and career development affects different individuals differently. According to identity threat theory, discretionary or non-discretionary responses occur when discrimination threatens one’s social identity and when this threat exceeds the coping resources available to the individual (Yang and Zhang, 2021). Depending on factors such as collective representations, situational cues, and individual characteristics, different people have different coping attitudes and present reactions when they perceive discrimination (Valentine et al., 1999). Therefore, it is not the perception of discrimination that affects the career exploration of higher vocational students, but the perception of discrimination reaches a certain level and ultimately affects individual career exploration behavior through specific internal mechanisms. There may be a mediating role in the mechanism of action between discrimination perception and career exploration. Core self-evaluation is a kind of overall self-evaluation, which is a potential and broad personality structure, which is defined as the most basic evaluation and estimation of the individual’s abilities and values (Judge, 1997). According to the intrinsic psychological mechanism of core self-evaluation-motivation mechanism, individuals with high core self-evaluation have higher level of task motivation because they believe more in their own ability level. Therefore, when vocational college students perceive employment discrimination in the job market, individuals with high core self-evaluation have more positive self-concept, they are more confident in their ability level, providing motivation for career exploration. According to learned helplessness theory, external discriminatory events are internalized by individuals and act on them through discrimination perceptions, causing them to attribute failure to internal, pervasive, and stable uncontrollable causes, such as education, ability, etc., resulting in feelings of helplessness (Mikulincer, 1986). At this point, individuals with low levels of core self-evaluation become psychologically self-destructive and inactive in their actions, which discourages them from taking active mental and physical activities to achieve their career goals and reduces their own career exploration. Individuals with high levels of core self-evaluation are able to hold on to their commitment goals, believe they are capable of controlling and solving difficulties, and are still motivated to engage in active career exploration (Li and Nie, 2010; Judge and Kammeyer-Mueller, 2011). Existing studies have shown that perceived discrimination is negatively correlated with core self-evaluation (Shantz and Booth, 2014). Core self-evaluation is positively correlated with career exploration (Zhu et al., 2021). Therefore, according to the motivation mechanism of core self-evaluation and the view of learned helplessness theory, this study hypothesized that core self-evaluation plays a mediating role between perceived discrimination and vocational college Students’ career exploration.

### The mediating effect of psychological flexibility between discrimination perception and career exploration

According to the experience avoidance model, discrimination perception will deepen the individual’s cognition of their membership in vulnerable groups, and make individuals have a serious sense of helplessness (Hayes-Skelton and Eustis, 2020). Encourage individuals to use experience avoidance coping strategies to mitigate negative emotions from perceived discrimination (Masuda et al., 2009). Therefore, when higher vocational students perceive employment discrimination, they may have the mentality of avoiding employment, which is not conducive to individual career exploration behavior. Existing studies have shown that perceived discrimination is positively correlated with experience avoidance (Sassenberg et al., 2003). Psychological flexibility is the core content of acceptance and commitment therapy (ACT), one of the representatives of the third generation of cognitive behavioral therapy (Tyndall et al., 2020). Studies have suggested that psychological flexibility refers to that individuals focus on the present with an open and tolerant attitude under the guidance of a clear and meaningful value direction, rather than using the conceptualized past or experiential self to judge or let the ideas and concepts in the mind bind themselves, accept what cannot be changed or has occurred, and do meaningful or valuable things with committed actions (Yasinski et al., 2020). Several studies have shown that psychological flexibility can mitigate the negative effects of perceived discrimination, such as West et al. (2013) studies, which found that value clarification can alleviate the pressure of racial discrimination (Graham et al., 2013; West et al., 2013; Brown-Iannuzzi et al., 2014) survey showed that psychological flexibility moderates symptoms of depression and anxiety caused by experience of discrimination (Graham et al., 2013; Brown-Iannuzzi et al., 2014). So, according to the experiential avoidance model, this study suggests that psychological flexibility can alleviate employment avoidance problem caused by discrimination perception of higher vocational students, promote higher vocational students to actively carry out career exploration behavior. Psychological flexibility plays a mediating role between discrimination perception and career exploration of higher vocational students.

### The chain mediating effect of core self-evaluation and psychological flexibility between discrimination perception and career exploration

Existing studies suggest that psychological flexibility also very much depends upon the precise configuration of personality traits in each individual (Kashdan and Rottenberg, 2010). The Big Five personality is a significant antecedent variable of psychological flexibility, which is significantly negatively related to the Neuroticism dimension and significantly positively related to the Responsibility dimension (Chen L. et al., 2022). Core self-evaluation as a personality structure, describes traits that are not adequately described by the Big Five and there is partial overlap between the two (Judge et al., 2003). Therefore, core self-evaluation is also an important factor influencing psychological flexibility. Based on this, this study considers that core self-evaluation and psychological flexibility play a chain mediating role between perceived discrimination and vocational college Students’ career exploration.

### Main purpose and hypothesis

While previous research has explored the relationship between perceived discrimination, core self-evaluation, psychological flexibility and career exploration, fewer studies have explored how discrimination perceptions affect career exploration through the mediating role of core self-evaluation and psychological flexibility. Based on the analysis of relevant theories, this paper conducts an empirical study on this issue to verify the following hypotheses:

Hypothesis 1: Discrimination perceptions significantly and negatively predict career exploration among higher vocational students (discrimination perception**→** career exploration).

Hypothesis 2: Core self-evaluation plays an mediating role between discrimination perception and career exploration (discrimination perception**→** core self-evaluation**→** career exploration).

Hypothesis 3: Psychological flexibility plays an mediating role between discrimination perception and career exploration (discrimination perception**→** psychological flexibility**→** career exploration).

Hypothesis 4: Core self-evaluation and psychological flexibility play a chain mediating role between discrimination perception and career exploration of higher vocational students (discrimination perception**→** core self-evaluation**→** psychological flexibility**→** career exploration).

## Materials and methods

### Participants

According to China’s Ministry of Education, the number of college graduates in China is expected to reach 10.76 million in 2022, up 1.67 million from 2021, a record high in scale and number, with the number of annual graduates exceeding the 10 million mark for the first time. In addition, in China’s job market, vocational college students have lower advantages in employment than students with ordinary education. Since data collection was conducted at one specific time, the nature of this study was cross-sectional. Using a convenience sampling method, an online questionnaire survey was conducted through higher education teachers to students in four higher education institutions in Jiangsu Province, China, with students agreeing to participate voluntarily, 948 questionnaires were distributed, and 893 valid questionnaires (94.20%) were collected and collated.

### Measures

All tests were conducted in Mandarin Chinese.

#### Discrimination perception scale

The discrimination perception questionnaire compiled by Shen et al. (2009) was used to measure the discrimination perception level of higher vocational students (Shen et al., 2009). The questionnaire includes two dimensions of individuals and groups, with a total of six items. The Likert 5—point scoring method is, ranging from 1 (completely inconsistent) to 5 (completely consistent). The higher the total score is, the stronger the level of discrimination perception of higher vocational students is. In this study, the Cronbach’s α coefficient of the discrimination perception scale was 0.938.

#### Core self-evaluation scale

Du et al. (2012) translated and revised the Core Self-Evaluation Scale (CSES) developed by Judge et al. (2003). The scale has strong reliability and validity, which greatly simplifies the measurement steps of core self-evaluation and is widely used (Judge et al., 2003; Du et al., 2012). This study adopts this scale to measure the core self-evaluation level of higher vocational students. The scale is a single dimension scale with 10 items. The Likert 5-point scoring method is used to measure the core self-evaluation level of higher vocational students from 1 (totally disagree) to 5 (totally agree). Among them, 2, 3, 5, 7, 8, and 10 are reverse scores. The higher the total score is, the higher the core self-evaluation level of students is. In this study, the Cronbach’s α coefficient of the CSES was 0.941.

#### Psychological flexibility scale

This study reflected individual psychological flexibility by measuring two dimensions, empirical avoidance and cognitive integration, and the higher the empirical avoidance and cognitive integration scores, the lower the individual’s psychological flexibility (Bond et al., 2013; Edwards, 2019).

Bond et al. (2011) developed the Acceptance and Action Questionnaire II (AAQ-II) to reflect individuals’ psychological flexibility by measuring their level of experiential avoidance. The questionnaire consists of seven questions and is scored on a 7-point Likert scale, with higher cumulative scores indicating higher levels of avoidance and lower psychological flexibility (Bond et al., 2011). In this study, Cao et al. (2013) revised the Chinese version of the Acceptance and Action Questionnaire, Version 2, which was the first psychological flexibility questionnaire introduced into China and is currently the most commonly used questionnaire to measure individual psychological flexibility in China (Cao et al., 2013). In this study, the Cronbach’s α coefficient of the Acceptance and Action Scale was 0.937.

Gillanders et al. (2014) developed the Cognitive fusion questionnaire (CFQ) to reflect individuals’ psychological flexibility by measuring their degree of cognitive fusion and cognitive dissociation (Gillanders et al., 2014). This study used the Chinese version of this scale revised by Zhang et al. (2014), which has nine questions containing one dimension of cognitive integration, using a Likert 7-point scale, and the higher the cumulative score, the heavier the individual’s cognitive integration and the lower the psychological flexibility (Zhang et al., 2014). In this study, the Cronbach’s α coefficient for the cognitive integration scale was 0.965.

#### Career exploration scale

Xu (2008) locally revised the Career Exploration Scale (CES) compiled by Stumpf et al. (1983) to form the Career Exploration questionnaire, which was used to investigate the career exploration of Chinese college students in recent 3 months and has good reliability and validity (Stumpf et al., 1983; Xu, 2008). This study uses this questionnaire to investigate the vocational exploration of higher vocational college students. The scale has 18 questions and contains four dimensions: environmental exploration, self-exploration, purpose-system exploration, and amount of information. A Likert 5-point scale was used, ranging from 1 (very little) to 5 (very much), with higher total questionnaire scores representing a greater degree of individual career exploration. In this study, the Cronbach’s α coefficient for the CES was 0.966.

### Data analysis

The data were subjected to descriptive statistics, reliability analysis and correlation analysis using SPSS 26.0, and chain mediated effects test using Amos 23.0.

## Results

### Common-method variance testing

With the help of SPSS26.0 statistical analysis software, Harman one-way test for the presence of common method bias was used. The results showed that there were six factors with eigenvalues greater than one, and the first factor had an explanatory rate of 39.335%, which was less than the critical criterion of 40%. Therefore, the research instruments in this study do not have serious common-method variance problems, although they were all completed by higher vocational students.

### Correlation analysis of major study variables

Correlation analyses were conducted on discrimination perceptions, core self-evaluation, psychological flexibility, and career exploration. The results showed that discrimination perception was significantly negatively correlated with core self-evaluation, psychological flexibility and career exploration (*r* = −0.487, −0.497, −0.326, *p* < 0.01), core self-evaluation was significantly positively correlated with psychological flexibility and career exploration (*r* = 0.518, 0.352, *p* < 0.01), and psychological flexibility was significantly positively correlated with career exploration (*r* = 0.386, *p* < 0.01) (Table 1).

**TABLE 1:** Means, standard deviations, and correlations.

Variable	M	SD	1	2	3	4
Discrimination perception	2.446	0.889	1			
Core Self-evaluation	3.432	0.746	−0.487**	1		
Psychological flexibility	3.637	1.246	−0.497**	0.518**	1	
Career exploration	3.217	0.706	−0.326**	0.352**	0.386**	1

**p < 0.01, N = 893.

### Multiple mediating analyses between variables

**FIGURE 1:**
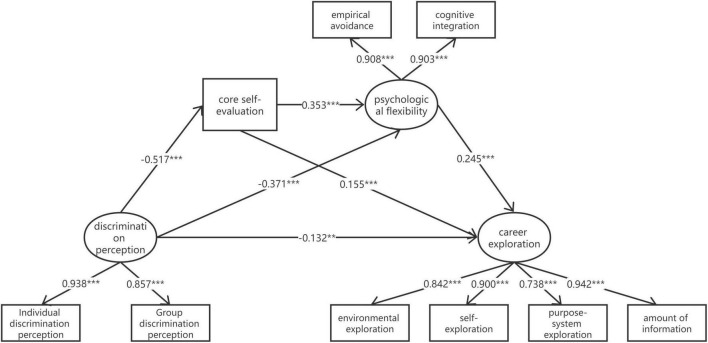
The influence path of perceived discrimination on career exploration. **p < 0.01, ***p < 0.001, N = 893.

The four variables of discrimination perception, core self-evaluation, psychological flexibility, and career exploration were significantly correlated with each other in two ways, satisfying the prerequisites for the test of mediating effects. Therefore, structural equation modeling was used to test for chain mediating effects in this study. The CSES was a single dimensional guide scale, explicit variables was used, discrimination perception, psychological flexibility, and career exploration contained multiple dimensions and latent variables were used. Data were analyzed using Amos 23.0, and the model fit met the critical values for each indicator: χ^2^/df = 2.481, TLI = 0.990, CFI = 0.994, GFI = 0.987, SRMR = 0.025, and RMSEA = 0.041, indicating a good model fit. The results of the path analysis showed that in the chain mediated effect model constructed in this study, discrimination perception and core self-evaluation (β = −0.517, *p* < 0.001), core self-evaluation and psychological flexibility (β = 0.353, *p* < 0.001), discrimination perception and psychological flexibility (β = −0.371, *p* < 0.001), and psychological flexibility and career exploration (β = 0.245, *p* < 0.001), discrimination perceptions and career exploration (β = −0.132, *p* < 0.01), and path coefficients between core self-evaluation and career exploration (β = 0.155, *p* < 0.001) were all statistically significant (Figure 1).

The mediating effect was tested using the Bootstrap method with 5,000 replicate samples and 95% confidence intervals were calculated, and the mediating effect was significant if the confidence interval did not contain 0 (Table 2). The mediating effect of core self-evaluation between discrimination perception and career exploration (discrimination perception**→** core self-evaluation career exploration) was −0.054, and the 95% confidence interval of the mediating effect was (−0.096, −0.020), indicating that the mediating effect of core self-evaluation was significant; The mediating effect of psychological flexibility between discrimination perception and career exploration (discrimination perception**→** psychological flexibility**→** career exploration) was −0.061, and the 95% confidence interval of the mediating effect was (−0.103, −0.028), indicating a significant mediating effect of psychological flexibility; The chain mediating effect of core self-evaluation and psychological flexibility between discrimination perception and career exploration (discrimination perception**→** core self-evaluation psychological flexibility**→** career exploration) was −0.030, and the confidence interval was (−0.047, −0.016), indicating that the chain mediation effect of core self-evaluation and psychological flexibility was significant.

**TABLE 2:** Effects and 95% confidence intervals for mode.

	Effect	LLCI	ULCI
Discrimination perception→core self-evaluation→career exploration	−0.054	−0.096	−0.020
Discrimination perception→psychological flexibility→career exploration	−0.061	−0.103	−0.028
Discrimination perception→core self-evaluation→psychological flexibility→career exploration	−0.030	−0.047	−0.016

## Discussion

In the field of organization and work, previous studies mainly focused on the impact of perceived discrimination on people’s work attitudes and work outcomes. This study shifts the perspective to higher education students before they engage in career behaviors and focuses on the effects of discrimination perceptions on their career exploration. Based on theoretical review and literature review, this paper constructs a chain mediation model to discuss the impact of perceived discrimination, core self-evaluation and psychological flexibility on vocational Students’ career exploration in China. The results support and verify the hypothesis of this study, and prove that core self-evaluation and psychological flexibility play a chain mediating role between perceived discrimination and career exploration. First, this study found that perceptions of discrimination can directly and negatively affect career exploration of higher vocational students, validating the first research hypothesis of this study. Through reviewing the existing literature, it is found that no study has directly verified the relationship between perceived discrimination and individual career exploration. Career exploration refers to a psychological or physical activity undertaken by individuals to achieve career goals, according to the career choice model in Social Cognitive Career Theory (SCCT), the first step of career choice is to express preliminary career goals, the second step is to take action to achieve goals, and the third step is to obtain performance achievements and form a feedback loop (Lent et al., 2000). In this process, the external environment will have a certain influence on individual career choice behavior, employment discrimination belongs to one of the environmental factors, when external employment discrimination is internalized by individuals, it affects individuals through discrimination perception, which will affect higher vocational Students’ career choice and career exploration.

This is similar to previous studies, previous studies have shown that high levels of discrimination perceptions have many negative effects on individuals’ career development (Sharma, 1983; Dispenza et al., 2012), this study confirms that high levels of discrimination perceptions are also detrimental to individuals’ career exploration. The job market in many developing countries prefers “highly educated” graduates with general education due to the “higher education boom” and “education level-oriented.” When higher vocational students perceive such unfair phenomenon, they may lack of motivation for career exploration due to helplessness or avoidance, leading to the decline of career exploration level. Therefore, every country should continue to vigorously promote the development of the typology of vocational institutions, strengthen the quality and excellence of vocational institutions, and improve the image of vocational institutions. Transform the stereotypical impressions of the public and the job market about vocational school students, enhance the attractiveness of vocational education and the self-confidence of higher vocational students, so as to reduce the level of discrimination perception of higher vocational students and promote the improvement of career exploration of this group.

Second, the results of the mediating role analysis showed that core self-evaluation partially mediated the relationship between discrimination perception and career exploration of higher vocational students, which verified the second research hypothesis of this study. According to the motivational mechanism of core self-evaluation, individuals with high levels of core self-evaluation are more certain of their abilities and react more constructively to negative feedback (Bono and Colbert, 2005). Individuals with high levels of core self-evaluation are able to set reasonable career goals and actively explore their careers, while individuals with low levels of core self-evaluation tend to pursue avoidant or protective goals, which are not conducive to career exploration (Bono and Judge, 2003). Therefore, counselors in Chinese higher vocational institutions should pay targeted attention to various aspects of Students’ career behaviors according to the different personality tendencies of different higher vocational students. For students with low levels of core self-evaluation, they should help them set reasonable career goals and encourage them to actively explore their careers on the basis of reasonable goals.

Thirdly, the results of the mediation analysis showed that psychological flexibility partially mediated the relationship between discrimination perception and career exploration of higher vocational students, which verified the third research hypothesis of this study. That is, psychological flexibility is a protective factor for the discrimination perceptions of higher vocational students and can significantly mitigate the negative effects of discrimination perceptions on individuals’ career exploration, which is similar to the results of previous studies (Judge and Bono, 2001; Kirikkanat, 2022). Students with high levels of psychological flexibility are less likely to conceptualize or experience stereotypes of vocational student identity, less likely to develop a state of avoidance when perceiving external discrimination, and more willing to accept the present situation and take positive action to do something more meaningful. Psychological flexibility is a core component of ACT and is a skill that can be developed through intervention (Harris and Samuel, 2020). Therefore, ACT should be incorporated into the career planning curriculum for higher vocational students to enhance psychological flexibility and promote active career exploration.

Finally, the results of the chain mediation analysis showed that discrimination perception could influence career exploration of higher vocational students through the chain mediation effect of core self-evaluation and psychological flexibility, which verified the fourth research hypothesis of this study. Core self-evaluation and psychological flexibility were significantly and positively correlated, i.e., the higher the level of core self-evaluation, the stronger the individual’s psychological flexibility. According to the identity threat theory, when discrimination threatens the social identity of higher vocational students, and the threat exceeds the coping resources of individuals, it will produce casual or non-casual reactions, which will lower the level of core self-evaluation of individuals, thus affecting the psychological flexibility of higher vocational students, and eventually decreasing their career exploration level. Previous studies have confirmed that core self-evaluation and psychological flexibility as mediating variables can significantly mitigate the negative effects of discrimination perception (Wagstaff et al., 2015; Liu et al., 2021). No studies have explored the chain mediating effect of core self-evaluation and psychological flexibility in discrimination perception and individual career development (discrimination perception**→** core self-evaluation**→** psychological flexibility**→** career exploration). In conclusion, this study verifies the hypothesis model of this study.

## Conclusion

Career exploration is a mental or physical activity that individuals adopt to achieve their career goals, and it has a significant role in promoting individual career behaviors and career abilities. In the context of the typological reform and development of vocational education in China, this study explored the relationship between discrimination perception and career exploration among Chinese higher vocational students. It was found that discrimination perception not only directly influenced career exploration of higher vocational students, but also indirectly influenced career exploration of higher vocational students through the separate mediation of core self-evaluation and psychological flexibility and the chain mediation of core self-evaluation and psychological flexibility. In conclusion, discrimination perception is not conducive to career exploration, and core self-evaluation and psychological flexibility can significantly promote career exploration of higher vocational students.

## Limitations

There are some limitations of this study. First, the cross-sectional survey could not observe changes in the longitudinal relationship between discrimination perceptions and career exploration of higher vocational students, and future research could use a longitudinal design to examine specific causal relationships between variables. Second, there are other variables not included in this study that may have an impact on career exploration of higher vocational students, such as family environment, personal traits, and other factors, and the lack of these unobserved variables may have an impact on the estimates reported in this study. Finally, the subjects of this study were students from four higher vocational institutions in Jiangsu Province. Jiangsu Province is one of the more economically and culturally developed provinces in China. “Jiangsu should strive to be at the forefront of deepening reforms across the board,” and in recent years, Jiangsu Province has been at the forefront of pilot reforms in the country, whether in economic construction, political and cultural construction, or educational reform. Especially in education, Jiangsu Province is a pilot STEM education program for primary and secondary schools, a pilot 1+X certificate system, and the first pilot province of the new college entrance examination reform. Therefore, it is somewhat one-sided to investigate only the higher vocational students in Jiangsu Province.

## Data availability statement

The original contributions presented in the study are included in the article/supplementary material, further inquiries can be directed to the corresponding authors.

## Ethics statement

This study involving human participants was reviewed and approved by the Ethics Committee of Nanjing Normal University. The ethics committee waived the requirement of written informed consent for participation.

## Author contributions

XL put forward the core point of the research and wrote the manuscript, and supervised the topic selection and research design. XL and XS participated in writing and data analysis. QH was responsible for data collection and modifying the manuscript. All authors contributed to the article and approved the submitted version.

## References

[B1] Atkinson A. B. Hills J. (1998). *Exclusion, Employment and Opportunity. LSE STICERD Research Paper no. CASE004.* London: LSE STICERD.

[B2] Blien U. Sanner H. (2014). Technological progress and employment. *Econ. Bull.* 34 245–251.

[B3] Bond F. W. Hayes S. C. Baer R. A. Carpenter K. M. Guenole N. Orcutt H. K. (2011). Preliminary psychometric properties of the Acceptance and Action Questionnaire–II: A revised measure of psychological inflexibility and experiential avoidance. *Behav. Ther.* 42 676–688. 10.1016/j.beth.2011.03.007 22035996

[B4] Bond F. W. Lloyd J. Guenole N. (2013). The work-related acceptance and action questionnaire: Initial psychometric findings and their implications for measuring psychological flexibility in specific contexts. *J. Occup. Organ. Psychol.* 86 331–347. 10.1111/joop.12001

[B5] Bono J. E. Colbert A. E. (2005). Understanding responses to multi-source feedback: The role of core self-evaluations. *Pers. Psychol.* 58 171–203. 10.1111/j.1744-6570.2005.00633.x

[B6] Bono J. E. Judge T. A. (2003). Core self-evaluations: A review of the trait and its role in job satisfaction and job performance. *Eur. J. Personal.* 17:S5–S18. 10.1002/per.481

[B7] Brown-Iannuzzi J. L. Adair K. C. Payne B. K. Richman L. S. Fredrickson B. L. (2014). Discrimination hurts, but mindfulness may help: Trait mindfulness moderates the relationship between perceived discrimination and depressive symptoms. *Personal. Individ. Differ.* 56 201–205. 10.1016/j.paid.2013.09.015 24347755 PMC3862075

[B8] Cao J. Ji Y. Zhu Z. H. (2013). Reliability and validity of the Chinese version of the Acceptance and Action Questionnaire-(AAQ-II) in college students. *Chinese Mental Health J.* 27 873–877.

[B9] Chan C. C. (2018). The relationship among social support, career self-efficacy, career exploration, and career choices of Taiwanese college athletes. *J. Hosp. Leis. Sport Tour. Educ.* 22 105–109. 10.1016/j.jhlste.2017.09.004

[B10] Chen H. Liu F. Wen Y. (2022). The influence of college students’ core self-evaluation on job search outcomes: chain mediating effect of career exploration and career adaptability. *Curr. Psychol.* 10.1007/s12144-022-02923-4 [Epub ahead of print]. 35228786 PMC8865730

[B11] Chen L. Qu L. Hong R. Y. (2022). Pathways Linking the Big Five to Psychological Distress: Exploring the Mediating Roles of Stress Mindset and Coping Flexibility. *J. Clin. Med.* 11:2272. 10.3390/jcm11092272 35566398 PMC9105170

[B12] Chen S. Xue Y. Chen H. Ling H. Wu J. Gu X. (2021). Making a Commitment to Your Future: Investigating the Effect of Career Exploration and Career Decision-Making Self-Efficacy on the Relationship between Career Concern and Career Commitment. *Sustainability* 13:12816.

[B13] Dispenza F. Watson L. B. Chung Y. B. Brack G. (2012). Experience of career-related discrimination for female-to-male transgender persons: A qualitative study. *Career Dev. Q.* 60 65–81. 10.1002/j.2161-0045.2012.00006.x

[B14] Du J. Z. Zhang X. Zhao Y. (2012). Reliability, validation and construct confirmatory of core self-evaluations scale. *Psychol. Res.* 5 54–60.

[B15] Edwards D. J. (2019). Age, pain intensity, values-discrepancy, and mindfulness as predictors for mental health and cognitive fusion: hierarchical regressions with mediation analysis. *Front. Psychol.* 10:517. 10.3389/fpsyg.2019.00517 30899236 PMC6416201

[B16] Gillanders D. T. Bolderston H. Bond F. W. Dempster M. Flaxman P. E. Campbell L. (2014). The development and initial validation of the cognitive fusion questionnaire. *Behav. Ther.* 45 83–101. 10.1016/j.beth.2013.09.001 24411117

[B17] Graham J. R. West L. M. Roemer L. (2013). The experience of racism and anxiety symptoms in an African-American sample: Moderating effects of trait mindfulness. *Mindfulness* 4 332–341. 10.1007/s12671-012-0133-2

[B18] Harnois C. E. (2015). Race, ethnicity, sexuality, and women’s political consciousness of gender. *Soc. Psychol. Q.* 78 365–386. 10.1177/0190272515607844

[B19] Harris E. Samuel V. (2020). Acceptance and commitment therapy: A systematic literature review of prevention and intervention programs for mental health difficulties in children and young people. *J. Cogn. Psychother.* 34 280–305. 10.1891/JCPSY-D-20-00001 33372124

[B20] Hayes-Skelton S. A. Eustis E. H. (2020). “Experiential avoidance,” in *Clinical Handbook of Fear and Anxiety: Maintenance Processes and Treatment Mechanisms*, eds Abramowitz J. S. Blakey S. M. (Washington, D.C: American Psychological Association), 115–131. 10.1037/0000150-007

[B21] Hu S. Hood M. Creed P. A. Shen X. (2020). The relationship between family socioeconomic status and career outcomes: A life history perspective. *J. Career Dev.* 49 600–615. 10.1177/0894845320958076

[B22] Jordaan J. P. (1977). Career development theory. *Int. Rev. Appl. Psychol.* 26 107–114. 10.1111/j.1464-0597.1977.tb01072.x

[B23] Judge T. A. (1997). The dispositional causes of job satisfaction: A core evaluations approach. *Res. Organ. Behav.* 19 151–188.

[B24] Judge T. A. Bono J. E. (2001). Relationship of core self-evaluations traits—self-esteem, generalized self-efficacy, locus of control, and emotional stability—with job satisfaction and job performance: A meta-analysis. *J. Appl. Psychol.* 86:80. 10.1037/0021-9010.86.1.80 11302235

[B25] Judge T. A. Erez A. Bono J. E. Thoresen C. J. (2003). The core self-evaluations scale: Development of a measure. *Personnel Psychol.* 56 303–331. 10.1111/j.1744-6570.2003.tb00152.x

[B26] Judge T. A. Kammeyer-Mueller J. D. (2011). Implications of core self-evaluations for a changing organizational context. *Hum. Res. Manag. Rev.* 21 331–341. 10.1016/j.hrmr.2010.10.003

[B27] Kashdan T. B. Rottenberg J. (2010). Psychological flexibility as a fundamental aspect of health. *Clin. Psychol. Rev.* 30 865–878. 10.1016/j.cpr.2010.03.001 21151705 PMC2998793

[B28] Kirikkanat B. (2022). The predictors of Turkish youth’s career planning: trait emotional intelligence, cognitive flexibility, and resilience. *Int. J. Educ. Vocat. Guid.* 10.1007/s10775-022-09538-y

[B29] Lent R. W. Brown S. D. Hackett G. (2000). Contextual supports and barriers to career choice: A social cognitive analysis. *J. Couns. Psychol.* 47 36–49. 10.1037/0022-0167.47.1.36

[B30] Li J. B. Nie Y. G. (2010). Reflection and prospect on core self-evaluations. *Adv. Psychol. Sci.* 18:1848.

[B31] Lindstrom L. Doren B. Metheny J. Johnson P. Zane C. (2007). Transition to employment: Role of the family in career development. *Except. child.* 73 348–366. 10.1177/001440290707300305

[B32] Liu X. Xie T. Li W. Tao Y. Liang P. Zhao Q. (2021). The relationship between perceived discrimination and wellbeing in impoverished college students: a moderated mediation model of self-esteem and belief in a just world. *Curr. Psychol.* 10.1007/s12144-021-01981-4

[B33] Masuda A. Price M. Anderson P. Schmertz S. K. Calamaras M. (2009). The role of psychological flexibility in mental health stigma and psychological distress for the stigmatizer. *J. Soc. Clin. Psychol.* 28 1244–1262. 10.1521/jscp.2009.28.10.1244

[B34] Mikulincer M. (1986). Attributional processes in the learned helplessness paradigm: Behavioral effects of global attributions. *J. Personal. Soc. Psychol.* 51:1248. 10.1037/0022-3514.51.6.1248 3806360

[B35] Pavlova M. K. Silbereisen R. K. (2014). Coping with occupational uncertainty and formal volunteering across the life span. *J. Vocat. Behav.* 85 93–105. 10.1016/j.jvb.2014.05.005

[B36] Sassenberg K. Kessler T. Mummendey A. (2003). Less negative= more positive? Social discrimination as avoidance or approach. *J. Exp. Soc. Psychol.* 39 48–58. 10.1016/S0022-1031(02)00519-X

[B37] Selleck-Harwell I. (2004). *Early Adolescents’ Career Explorations: Examination of Family, School, and Peer Influences.* Charlotte: Johnson & Wales University.

[B38] Shantz A. Booth J. E. (2014). Service employees and self-verification: The roles of occupational stigma consciousness and core self-evaluations. *Hum. Relat.* 67 1439–1465. 10.1177/0018726713519280

[B39] Sharma S. (1983). Women in Medicine: Traditional Biases and Emerging Trends. *Equal Opportunities Int.* 2 25–31. 10.1108/eb010390

[B40] Shen J. Hu X. Liu X. (2009). Left-over children’s perceived discrimination: its characteristics and relationship with personal wellbeing. *J. Henan Univer.* 49 116–121.

[B41] Stumpf S. A. Colarelli S. M. Hartman K. (1983). Development of the career exploration survey (CES). *J. Vocat. Behav.* 22 191–226. 10.1016/0001-8791

[B42] Suomi A. Schofield T. P. Butterworth P. (2020). Unemployment, employability and COVID19: how the global socioeconomic shock challenged negative perceptions toward the less fortunate in the Australian context. *Front. Psychol.* 11 27–45. 10.3389/fpsyg.2020.594837 33178089 PMC7593239

[B43] Super D. E. Jordaan J. P. (1973). Career development theory. *Br. J. Guid. Couns.* 1 3–16. 10.1080/03069887308259333

[B44] Super D. E. Knasel E. G. (1981). Career development in adulthood: Some theoretical problems and a possible solution. *Br. J. Guid. Couns.* 9 194–201. 10.1080/03069888108258214

[B45] Tilak J. B. (2003). “Vocational education and training in Asia,”. In Keeves J. P. Watanabe R. Maclean R. Renshaw P. D. Power C. N. Baker R. Gopinathan S. Kam H. W. Cheng Y. C. Tuijnman A. C. (eds.) *International Handbook of Educational Research in the Asia-Pacific Region.* 673–686. 10.1007/978-94-017-3368-7_46

[B46] Triana M. Wagstaff M. F. Colella A. (2010). Managing diversity: How organizational efforts to support diversity enhance affective commitment and reduce turnover intent for employees who experience discrimination at work. *Personnel Psychol.* 63 817–843. 10.1111/j.1744-6570.2010.01189.x

[B47] Tyndall I. Waldeck D. Pancani L. Whelan R. Roche B. Pereira A. (2020). Profiles of psychological flexibility: A latent class analysis of the acceptance and commitment therapy model. *Behav. Modification* 44 365–393. 10.1177/0145445518820036 30580551

[B48] Valdivia C. Flores L. Y. (2012). Factors affecting the job satisfaction of Latino/a immigrants in the Midwest. *J. Career Dev.* 39 31–49. 10.1177/0894845310386478

[B49] Valentine S. Silver L. Twigg N. (1999). Locus of control, job satisfaction, and job complexity: The role of perceived race discrimination. *Psychol. Rep.* 84 1267–1273. 10.2466/pr0.1999.84.3c.1267

[B50] Wagstaff M. F. del Carmen Triana M. Kim S. Al-Riyami S. (2015). Responses to discrimination: Relationships between social support seeking, core self-evaluations, and withdrawal behaviors. *Hum. Res. Manag.* 54 673–687. 10.1002/hrm.21634

[B51] Wang J. Fan W. Cheung F. M. Wang Q. Li M. (2019). Personality and Chinese adolescents’ career exploration: The mediation effects of self-efficacy and perceived parental support. *J. Pacific. Rim. Psychol.* 13:e28–e29. 10.1017/prp.2019.16

[B52] West L. M. Graham J. R. Roemer L. (2013). Functioning in the face of racism: Preliminary findings on the buffering role of values clarification in a Black American sample. *J. Contextual Behav. Sci.* 2 1–8. 10.1016/j.jcbs.2013.03.003

[B53] Xu C. (2008). *Career Decision-Making Self Efficacy and its Relationship to Anxiety and Career Exploration (in Chinese).* Ph.D thesis. Wuhan: Central China Normal University.

[B54] Yang N. Zhang R. (2021). Under threat: emotional and behavioral responses to occupational identity threat. *J. Manag. Organ.* 1–17. 10.1017/jmo.2021.53

[B55] Yang T. C. Chen I. C. Choi S. W. Kurtulus A. (2019). Linking perceived discrimination during adolescence to health during mid-adulthood: Self-esteem and risk-behavior mechanisms. *Soc. Sci. Med.* 232 434–443. 10.1016/j.socscimed.2018.06.012 30025883 PMC7416483

[B56] Yasinski C. Hayes A. M. Ready C. B. Abel A. Görg N. Kuyken W. (2020). Processes of change in cognitive behavioral therapy for treatment-resistant depression: psychological flexibility, rumination, avoidance, and emotional processing. *Psychother. Res.* 30 983–997. 10.1080/10503307.2019.1699972 31822203

[B57] Zhang W. Ji Y. Li X. Guo H. Zhu Z. (2014). Reliability and validity of the Chinese version of the Cognitive Fusion Questionnaire. *Chinese Mental Health J.* 28 40–44.

[B58] Zhu H. Zhang H. Tu A. Zhang S. (2021). The mediating roles of Core self-evaluation and career exploration in the association between proactive personality and job search clarity. *Front. Psychol.* 12:609050. 10.3389/fpsyg.2021.609050 PMC8211878 34149503

